# Preparation of curcumin self-micelle solid dispersion with enhanced bioavailability and cytotoxic activity by mechanochemistry

**DOI:** 10.1080/10717544.2017.1422298

**Published:** 2018-01-05

**Authors:** Qihong Zhang, Nikolay E. Polyakov, Yulia S. Chistyachenko, Mikhail V. Khvostov, Tatjana S. Frolova, Tatjana G. Tolstikova, Alexandr V. Dushkin, Weike Su

**Affiliations:** ^a^ National Engineering Research Center for Process Development of Active Pharmaceutical Ingredients, Collaborative Innovation Center of Yangtze River Delta Region Green Pharmaceuticals, Zhejiang University of Technology Hangzhou PR China; ^b^ Institute of Chemical Kinetics and Combustion Novosibirsk Russia; ^c^ Institute of Solid State Chemistry and Mechanochemistry Novosibirsk Russia; ^d^ N.N. Vorozhtsov Institute of Organic Chemistry SB RAS Novosibirsk Russia; ^e^ Novosibirsk State University Novosibirsk Russia; ^f^ Institute of Cytology and Genetics SB RAS Novosibirsk Russia; ^g^ Key Laboratory for Green Pharmaceutical Technologies and Related Equipment of Ministry of Education, College of Pharmaceutical Sciences, Zhejiang University of Technology Hangzhou PR China

**Keywords:** Mechanical ball milling, solid dispersion, curcumin, self-micelle, cytotoxic activity, bioavailability

## Abstract

An amorphous solid dispersion (SD) of curcumin (Cur) with disodium glycyrrhizin (Na_2_GA) was prepared by mechanical ball milling. Curcumin loaded micelles were self-formed by Na_2_GA when SD dissolved in water. The physical properties of Cur SD in solid state were characterized by differential scanning calorimetry, X-ray diffraction studies, and scanning electron microscope. The characteristics of the sample solutions were analyzed by reverse phase HPLC, UV–visible spectroscopy, ^1^H NMR spectroscopy, gel permeation LC, and transmission electron microscopy. *In vitro* cytotoxic tests demonstrated that Cur SD induced higher cytotoxicity against glioblastoma U-87 MG cells than free Cur. Besides, an improvement of membrane permeability of Cur SD was confirmed by parallel artificial membrane permeability assay. Further pharmacokinetic study of this SD formulation in rat showed a significant ∼19-fold increase of bioavailability as comparing to free Cur. Thus, Cur SD provide a more potent and efficacious formulation for Cur oral delivery.

## 1. Introduction

**Figure 1.**
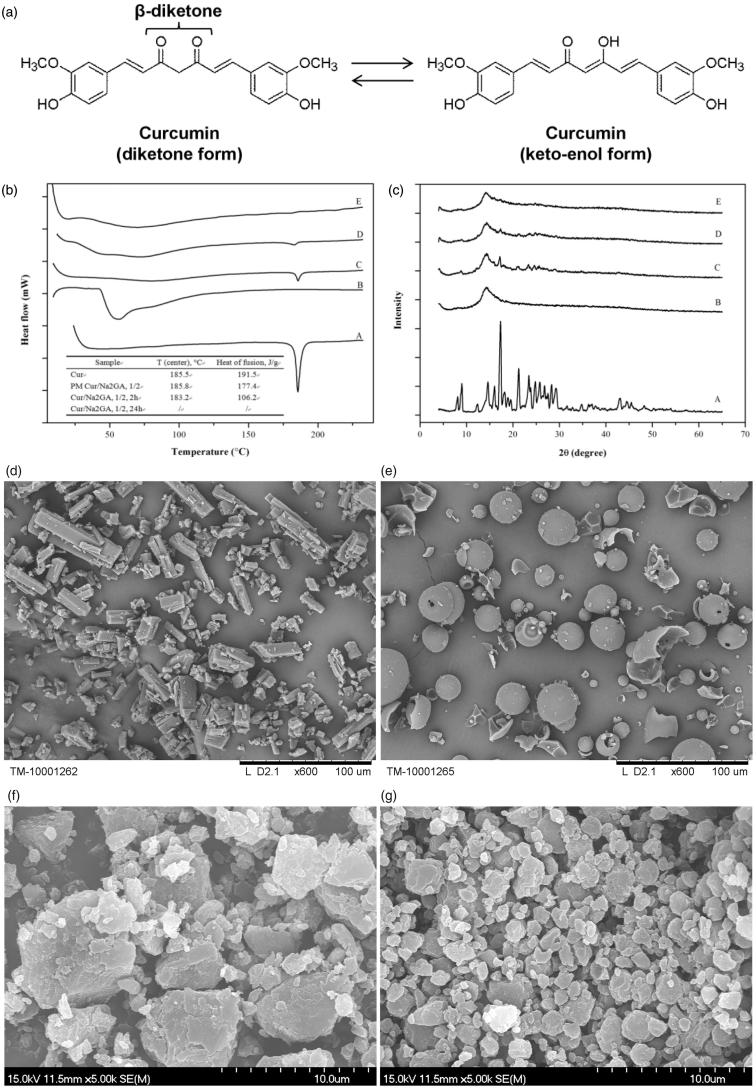
(a) Structure and tautomerism of curcumin molecules; (b) DSC thermograms and (c) X-ray diffractograms of: A: curcumin; B: Na_2_GA; C: physical mixture (PM); D: Cur SD (molar ratio 1/2, milling 2 h); E: Cur SD (molar ratio 1/2, milling 24 h); electron micrographs of: (d) curcumin; (e) Na_2_GA; (f) Cur SD (molar ratio 1/2, milling 2 h); (g) Cur SD (molar ratio 1/2, milling 24 h).

Curcumin (Cur) is a natural diphenolic compound derived from *Curcuma longa* L. (Curcuma domestica Valeton). Curcumin is an oil soluble pigment, practically insoluble in water at acidic and neutral pH, soluble in alkali. It is stable at high temperatures and in acids, but unstable in alkaline conditions and in the presence of light (Priyadarsini, 2014). The principal coloring components of Cur exhibit a keto-enol tautomerism (Figure 1(a)).

In addition to its use as a food additive and spice, Cur possesses numerous health beneficial effects such as anticarcinogenic (Piper et al., 1998; Fang et al., 2005), antimicrobial, antiviral (Moghadamtousi et al., 2014), immunomodulatory (Gao et al., 2001; Varalakshmi et al., 2008; Srivastava et al., 2011), anti-inflammatory, and antioxidant properties (Motterlini et al., 2000). Due to the pharmacological efficacy and safety, Cur has been investigated extensively in a wide range of research areas. However, the oral bioavailability of hydrophobic Cur is low due to its poor solubility in water and the instability under physiological and alkaline pH conditions, and thus only a small fraction of ingested Cur can be absorbed (Kocher et al., 2015). Various formulations have been developed to improve its aqueous solubility and oral bioavailability, such as nanoparticle (Bisht et al., 2007; Anand et al., 2010; Madane & Mahajan, 2016; Taki et al., 2016), liposome (Li et al., 2005, 2007; Wan et al., 2016), micelles (Gong et al., 2013; Yoncheva et al., 2015; Phan et al., 2016; Kumari et al., 2017), phospholipids complex (Maiti et al., 2007; Kumari et al., 2017), and solid dispersions (SDs) (Paradkar et al., 2004; Seo et al., 2012). Among above methods, some need expensive excipients (e.g. lipids, cholesterol, etc.), long preparation time, complicated procedures, various organic solvents (ethanol, dichloromethane, dimethyl sulfoxide, etc.) which need heating, vacuum evaporation or freeze-drying process to eliminate the solvent. It will increase the risk and cost of production. Amorphous SD can be defined as molecular mixtures of poor water soluble drugs with hydrophilic carriers, responsible for modulating drug release profile, and characterized by the reduction of drug particle size to a molecular level solubilizing or co-dissolving the drug in the soluble carriers (Vasconcelos et al., 2016). Solid dispersion currently represents the most exciting research and development field related to amorphous pharmaceutical products. Two major distinct processes are widely used to prepare amorphous materials: solvent evaporation and melting. However, in the case of solvent evaporation method, organic solvents may be difficult to be removed from the final product, which can be especially concerning when highly toxic solvents are required to be employed. The major drawback of melting processes is high temperatures that may induce drug degradation or decomposition. In addition, melting processes require drug solubility/miscibility, which can be very difficult to achieve for some hydrophobic molecules. To the best of our knowledge, there are few studies on SD of Cur prepared by mechanical ball milling process. Recently, mechanochemistry has become an important subject of interest in pharmaceutical sciences for its role in the development of green synthesis (Wang, 2013; Yu et al., 2016), supramolecular structures (Dushkin & Tolstikova, 2012), cocrystal synthesis (Braga et al., 2013), and amorphous SDs (Chistyachenko et al., 2015). When a solid molecular compound is subjected to high energy mill, its structural and microstructural characters change considerably as well as its physical and chemical stability. All these changes may have a significant impact on biopharmaceutical properties, e.g. enhanced solubility and bioavailability (Descamps & Willart, 2016). No solvent or high temperature is needed during the process of mechanochemical treatment.

Glycyrrhizic acid (GA) is a triterpene glycoside extracted from licorice root and demonstrates antiviral, anti-inflammatory, and anticancer properties (Su et al., 2017). Due to its amphiphilic property, GA is capable of forming complexes with a variety of hydrophobic molecules, substantially increasing their solubility and enhancing the permeability of drug through cell membranes (Selyutina et al., 2016a). In addition, disodium glycyrrhizin (Na_2_GA) is the salt of GA, which can undergo hydrolysis in aqueous solutions and formed a free GA. Its advantage is that the formation of solution is with lower viscosity in contrast with GA solutions. We can expect the synergetic effect of using Na_2_GA as a drug delivery system for Cur. Given that mechanochemical technology and Na_2_GA have the potential to improve the bioavailability of water insoluble drugs, we then evaluate bioavailability and bio-efficacy of Cur as an amorphous SD in a matrix consisting of Na_2_GA.

## 2. Materials and methods

### 2.1. Materials

Curcumin was purchased from Xi’an Jinheng Chemical Co. Ltd. (Xian, China, purity: 98%, melting point: 185.51 °C). Disodium salt of glycyrrhizic acid (Na_2_GA) was purchased from Shaanxi Pioneer Biotech Co., Ltd. (Xian, China, purity: 98%). Dulbecco’s modified eagle medium (DMEM) and fetal bovine serum (FBS) were purchased from Gibco (Carlsbad, CA). Penicillin-streptomycin, β-glucuronidase/sulfatase (EC 3.2.1.31) from Helix pomatia was purchased from Sigma (St. Louis, MO). All other chemicals used were of analytical grade.

### 2.2. Preparation of solid dispersion by mechanochemical treatment

The roll mill VM-1 (accelerated speed of 1 g) was used to prepare SD. Briefly, 6.56 g Cur and 15.44 g Na_2_GA (molar ratio 1/1), or 3.86 g Cur and 18.14 g Na_2_GA (molar ratio 1/2), or 2.11 g Cur and 19.89 g Na_2_GA (molar ratio 1/4) were added to 300 mL vial with 660.0 g steel balls (diameter 22 mm) with milling time of 24 h, rotation speed 157 rpm and samples were picked out at 2, 4, 8, 16 h, and 24 h, respectively.

### 2.3. Analysis of curcumin by HPLC

The amount of Cur was determined by using a high performance liquid chromatography (HPLC) system (Agilent 1200, Santa Clara, CA) with column Zorbax Eclipse XDB-C_18_, 4.6 × 50 mm at +30 °C and diode-array detector. Acetonitrile–acetate buffer (55:45) was used as eluent (pH = 3.4) with flow rate of 1.0 mL/min, and detection wavelength was 430 nm.

### 2.4. Content test for curcumin in solid dispersion

To determine the content of Cur in SD after mechanical treatment, the weighted powder samples were dissolved in 25 mL of a mixture solution (distilled water to ethanol, 1:1, v/v), respectively. In all cases, all components of SD were completely dissolved, and then analyzed by HPLC.

### 2.5. Differential scanning calorimetry (DSC)

Thermal analysis of the samples was carried out by DSC with the DSC-550 instrument (Instrument Scientific Specialists Inc., Omaha, NE) in Ar atmosphere. Temperature program: 20–250 °C. Heating rate: 10 °C/min.

### 2.6. Powder X-ray diffraction (XRD)

X-ray diffraction analysis of solid complexes was carried out on a DRON-4 equipment (‘Burevestnik’, St. Petersburg, Russia) using CuK*α* radiation, counter speed 2°/min, range of intensity measurement – 1000.

### 2.7. Scanning electron microscopy (SEM)

Electronic images were acquired using a Hitachi TM-1000 microscope (Tokyo, Japan). Coating of samples with gold was performed using a JEOL JFC-1600 auto fine coater (Tokyo, Japan). The coating parameters were as follows: sputtering time 30 s, amperage 30 mA, and film thickness 15 nm.

### 2.8. UV–vis spectroscopy

The sample solutions were detected by UV-VIS spectroscopy (UV-1800, Shimadzu, Kyoto, Japan). Ten milligrams of pure Cur, Cur SD were dissolved in 5 mL of different solvents (including water) and filtered. The Cur concentrations of complex solutions were detected by HPLC.

### 2.9. ^1^H NMR spectroscopy

^1^H NMR spectra were recorded on Bruker Avance III 500 MHz spectrometer, and the spin–spin relaxation time *T*_2_ was measured using the Carr-Purcell–Meiboom-Gill (CPMG) pulse sequence from Avance version of Bruker pulse sequence library: P_1_ (90°) – (*τ* – P_2_ (180°) – *τ*)*n* – registration, where *τ* = 0.5 ms – fixed time delay, and *n* varied from 0 to 2000. The compositions prepared mechanochemically and by physical mixing of components were investigated in D_2_O and CD_3_OD (Aldrich, St. Louis, MO, 99.8%) solutions as well as in their mixture. Spin–spin relaxation times (*T*_2_) are sensitive to molecular motions. The *T*_2_ value is closely related to the mobility of a molecule and is inversely proportional to the rotational correlation time. Thus, using *T*_2_ data, one can probe changes in the environment or state (free/bound) of the molecules (Deese et al., 1982).

### 2.10. Solubility determination

To determine the solubility, excess samples were dissolved in 10 mL of distilled water with stirring in orbital shaker (200 rpm) for 12 h at +37 °C. At last, sample solutions were filtered and analyzed by HPLC.

### 2.11. Dissolution determination

Dissolution tests of pure Cur and SD samples were performed in a dissolution tester (HTY-EU802, Hangzhou, China) at the paddle rotation speed of 100 rpm in 900 mL of pH 1.2 simulated gastric media and 6.8 phosphate buffer maintained at 37 ± 0.5 °C. Each formulation equivalent to 90 mg of Cur was put into dissolution vessel. At the predetermined time intervals, 3 mL of the sample was withdrawn and the equal volume of fresh medium was added into dissolution vessel. The collected samples were filtered through regenerated cellulose syringe filters. Initial sample volume of 2 mL was discarded, and then final 1 mL was suitably diluted with ethanol. Then, samples were analyzed by HPLC.

### 2.12. Molecular weight distribution

The molecular weight distribution (MWD) of the samples was investigated by gel permeation chromatography (GPC) on the Agilent 1200 chromatograph with column PL aquel-OH 40 (Santa Clara, CA), 300 × 7.5 mm column at 30 °C with refractometric detector. The solvent used was 0.02% NaN_3_ aqueous solution and flow rate was 1 mL/min. The calibration was performed by standard dextrans with molecular weights of 1, 5, 12, 25, 80, 150, 270, and 410 kDa. Agilent GPC data analysis program was used to process the results (Santa Clara, CA).

### 2.13. Particle characterization and zeta potential

Hydrodynamic diameter, polydispersity index (PDI), and zeta potential of micelle were determined by the Photon Correlation Spectroscopy (PCS) machine and electrophoretic mobility titration (Nano ZS 90 nanoseries, Malvern Instruments Ltd., Malvern, UK). Samples (10 mg) were diluted with 10 mL of filtered distilled water to eliminate the effect of viscosity caused by the ingredients. Hydrodynamic diameter (based on volume measurement), PDI, and zeta potential were obtained from the average of three measurements at 25 °C.

### 2.14. Transmission electron microscopy (TEM)

The morphology of micelle was observed under TEM. The samples were prepared by the negative staining method with aqueous uranyl acetate as the staining agent. One drop of sample was placed on the carbon Formvar-coated cooper grid (200 mesh) for 5 min, and then the excess solution was wiped away with filter paper to form a thin liquid film in the copper grid. Next, one drop of aqueous uranyl acetate was placed on the copper grid. The excess liquid was also wiped away with filter paper. The samples were dried in air before being visualized with a JEOL JEM-1230 (HR) transmission electron microscope at a working voltage of 80 kV (Tokyo, Japan).

### 2.15. Phase solubility study

Different amounts of complex in successively increased concentration (2, 4, 6, 8, 10, 12, 15, and 20 mM) were added to a 50 mL flat-bottomed flask with 10 mL of distilled water. Then, they were shaken in the orbital shaker with 200 rpm at +37 °C, the temperature of *in vivo* gastrointestinal tract. Until the equilibrium of solution was established, the solutions were separately filtered and determined by HPLC method. To explore the thermodynamics parameters of Cur complexation with Na_2_GA in aqueous solution, the same experiments were separately carried out at +25 °C, +30 °C, but unfortunately obtained results had large experimental errors. Stability constants (*K*_s_) and the thermodynamic values for the formation of supramolecular systems were calculated by the following equations:

(1) $$K_{\text{s}}\;=\frac{Slope}{S_{0}\times (1-S_{0})}$$

(2) ΔG=-R×T×In Ks

### 2.16. *In vitro* parallel artificial membrane permeability assay (PAMPA)

PAMPA was a method for predicting passive intestinal absorption (Kansy et al., 1998; Mccallum, 2013). The assay was carried out in a 12-well, Transwell inserts (polycarbonate membrane, 12 mm i.d., 0.4 μm pore size, 1.12 cm^2^ area, Corning Incorporated, Corning, NY). The ability of compounds to diffuse from a donor compartment into an acceptor compartment was evaluated, by placing a polycarbonate membrane filter pretreated with a model lipid containing organic solvent between the two compartments. The artificial membrane (Mccallum, 2013) was prepared by carefully pipetting 60 µL of the 5% (v/v) hexadecane in hexane solution to each of the wells of the donor plate. The plate was placed into a fume hood for one hour to ensure complete evaporation of the hexane. After the hexane had evaporated, 1.5 mL of water with 5% (v/v) DMSO was added to each of the wells of the acceptor plate. The hexadecane treated donor plate was then placed on top of the acceptor plate taking care that the underside of the membrane was completely in contact with the solution in each of the acceptor wells. Then, 0.5 mL of Cur containing donor solutions (knowing quantities of free Cur or Cur SD diluted in a solution of 5 mL DMSO in 100 mL of water) was added to each well of the donor plate. The plate was covered and incubated at +25 °C under shaking at 200 rpm and permeation was evaluated after 0.5, 1.0, 1.5, 2.0, 2.5, 3.0, and 3.5 h.

### 2.17. Cytotoxic activity

#### 2.17.1. Cell culture

Cancer cell lines U-87 MG (glioblastoma) and MCF-7 (breast cancer) were obtained from the ATCC (ATCC number HTB-14 and HTB-22, respectively). Immortalized human fibroblasts used as normal cells were kindly provided by Alexander G. Shilov (Institute of Cytology and Genetics SB RAS). The cells were maintained in DMEM plus 10% FBS, under a temperature of 37 °C, atmosphere of 5% of CO_2_ and 90% humidity.

#### 2.17.2. MTT test

MTT test was performed according to a standard procedure (Mosmann, 1983). Cells were seeded in a 96-well plate at a density of 5 × 10^3^ cells/well and after 24 h of adherence, the cells were treated with different concentration of test compounds and incubated for 48 h at 37 °C in an atmosphere of 5% CO_2_ in air. Untreated cells were used as negative control and doxorubicin was used as a positive control. After the incubation period, 10 μL MTT solution (3 mg/mL of PBS) was added to each well followed by incubation for 3 h under the condition mentioned above. Then, the MTT-containing medium was removed and the formazan crystals were dissolved with 100 μL of isopropanol. Absorbance was measured at 630 and 492 nm using a microplate reader (Thermo Scientific, Waltham, MA). The cell viability percentage was calculated using the following formula: Viability (%)=OD (treated cells)/OD (untreated cells) × 100, where OD was an optical density of solution.

### 2.18. Bioavailability study

The pharmacokinetic study was carried out in male Sprague Dawley rats (10 weeks of age, 250–260 g body weight, provided by the Zhejiang Academy of Medical Science, Hangzhou, China). All animal (used in this experiment) handling procedures were performed in strict compliance with the PR China legislation for the use and care of laboratory animals and were approved by the University Committee for Animal Experiments. The animals were fasted overnight (12 h) and had free access to drinking water throughout the experimental period. Free Cur and Cur SD were administered at oral doses of 150 mg/kg Cur/rats, a suspension in 0.25% carboxymethyl-cellulose (CMC) with 0.1% tween 80. To evaluate the concentration of Cur in plasma, blood was collected into heparinized Eppendroff tubes at different time points: 0.083, 0.25, 0.5, 1, 2, 4, 6, 8, 12, and 24 h post-dosing and centrifuged (Sigma Laboratory Centrifuge, Model 3K-30, Osterode am Harz, Germany) at 9391 × *g* for 5 min to separate the plasma. The plasma samples were stored at –80 °C until further analysis.

#### 2.18.1. Preparation of samples for analysis

A 100 μL aliquot of plasma was transferred to a clean microcentrifuge tube and next treated with 100 μL of a solution containing 1000 U of *β*-glucuronidase in 0.1 M phosphate buffer (pH 6.8). The resulting mixture was then thoroughly vortexed and incubated at 37 °C for 1 h to hydrolyze the conjugated Cur. After incubation, 0.1 mL acetonitrile was added to 0.1 mL of plasma and centrifuged (Sigma Laboratory Centrifuge, Model3K-30, Osterode am Harz, Germany) at 9391 × *g* for 15 min, and then the supernatant was subjected to be analyzed by HPLC.

### 2.19. Statistical analysis

Standard pharmacokinetic parameters for free Cur and Cur SDs were calculated by linear trapezoidal method using the PKSolver, a freely available menu-driven add-in program for Microsoft Excel. The results were presented as mean ± standard error of the mean (SEM). One-way analysis of variance (ANOVA) was followed by Dunnett’s test for multiple comparisons statistical evaluation. *p* Values <.05 were considered significant.

## 3. Results and discussion

### 3.1. Physical characterization studies of Cur SD

The DSC thermograms of free Cur, physical mixtures (PMs), and Cur SD are shown in Figure 1(b). The related thermal parameters of peak melting temperature (*T*) and molar enthalpy (Δ*H*) are summarized in the inset. The DSC curves of free Cur exhibited endothermic peaks around 185 °C with ΔH value of 191.5 J g^−1^, which corresponded to its intrinsic melting points and suggested its high crystalline structure. However, the intensity of Cur peak was decreased significantly in Cur SD with milling time from 2 h to 24 h, as well as Δ*H* values decreased from 191.5 J g^−1^ to a minimal value that cannot be calculated, indicating that Cur had converted to an amorphous state and probably dispersed in molecular form in the bulk phase of carrier during mechanochemical process. X-ray diffractograms of free Cur, PM, and Cur SD are shown in Figure 1(c). Free Cur showed several characteristic peaks at 2*θ* angles within 30°, indicating its crystalline form. However, the characteristic crystalline peaks of Cur were significantly decreased in the diffractogram of Cur SD, and even disappeared in the 24 h milled Cur SD. The lack of the characteristic crystalline peaks and the decrease of endothermic peaks in the diffractogram and thermogram of Cur SD, respectively, were all consistent with the conversion of the formulation to a high energy amorphous dispersion.

The electron micrographs of initial substances and obtained Cur SD are shown in Figure 1(d–g). It could be clearly seen that the initial pure Cur and Na_2_GA had characteristic crystalline and intact shape. However, under mechanochemical milling processing, the destruction of the crystalline of Cur and spherical Na_2_GA particles occurred followed by the formation of polydisperse particles with irregular shape. As milling time was prolonged from 2 h to 24 h, the particle dispersed much more homogeneously, which might increase the surface area of the particle and therefore enhance the solubilization velocity.

### 3.2. Characteristics of Cur SD in solutions

In the recent article (Manolova et al., 2014), the tautomerism of Cur molecules in water/alcohol mixtures solvents was considered. On the other hand, some theoretical study indicated that Cur should have the self-assembling properties in water by varying the number of monomers both in parallel and anti-parallel orientation of the phenyl rings, which formed clusters up to 16 molecules of monomers (Hazra et al., 2014). To investigate the tautomerism and self-assembling of Cur in different solvents, UV–visible absorption spectra and ^1^H NMR relaxation technique were applied (Figure 2). Curcumin had the characteristic peak at wavelength of 431 nm when dissolved in 50–100% ethanol and 55% acetonitrile (HPLC condition). However, the maximum peak of Cur shifted from 431 nm to 340 nm when dissolved in water, probably indicating the shift in tautomers equilibrium from enol-keto to diketo tautomers. UV–visible absorption spectrum of Cur SD in different solvents is shown in Figure 2(b). Curcumin showed peak at wavelength of 431 nm at different ethanol concentration, and the sharpness of characteristic peak decreased and shoulder peak ranging from 350 to 400 nm and 450 to 500 nm turned up when dissolved in water. Any case, the intensive absorption at ∼340 nm indicated that Cur in its complexes/micelles with Na_2_GA existed mainly as enol-keto tautomer. Shoulder peaks in the spectra were likely to be associated with intermolecular interactions of Cur and Na_2_GA. Therefore, it could be concluded that molecules of Cur even in aqueous solutions of the associates with Na_2_GA were not in a polar environment and probably incorporated into micelles of Na_2_GA.

Figure 2.UV–visible absorption spectra of: (a) pure Cur and (b) Cur SD (molar ratio 1/2) in different solvents, A: water; B: 50% ethanol; C: 70% ethanol; D: 100% ethanol; E: 55% acetonitrile; (c) ^1^H NMR spectra of curcumin in different solvents; (d) dependence of *T*_2_ relaxation time (in logarithmic scale) of the curcumin protons (O-CH_3_) on the water/methanol ratio; (e) solubility and (f) dissolution profiles of Cur, physical mixtures (PM) and Cur SDs; (g) ^1^H NMR relaxation study: dependence of spin-echo decay (in logarithmic scale) and calculated *T*_2_ relaxation times of the curcumin protons (O-CH_3_) on the water/methanol ratio in the complex with Na_2_GA 1:4 ([Na_2_GA] = 1 mM). (h) TEM of Cur-Na_2_GA micelles; (i) phase solubility diagram of complex Cur/Na_2_GA in the aqueous solution at +37 °C.

**Figure:**
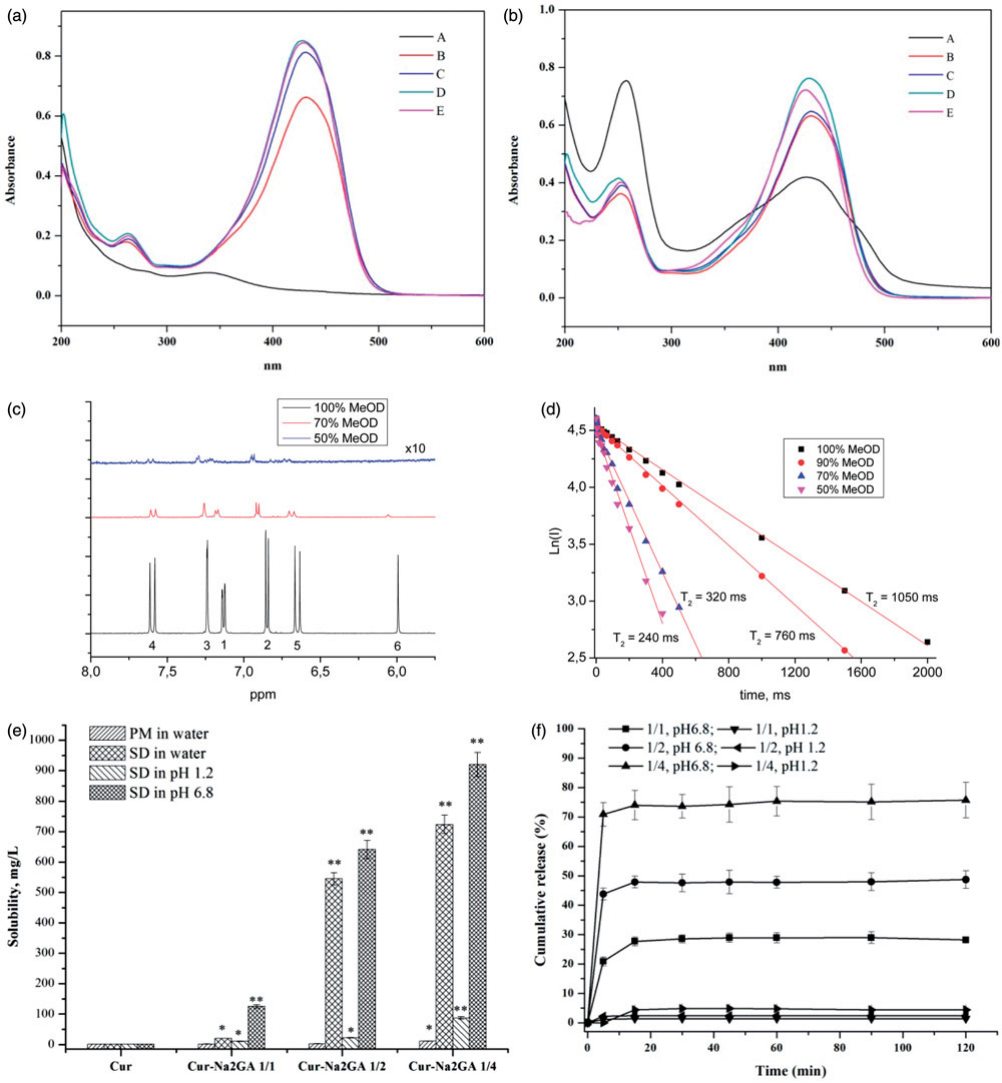

**Figure:**
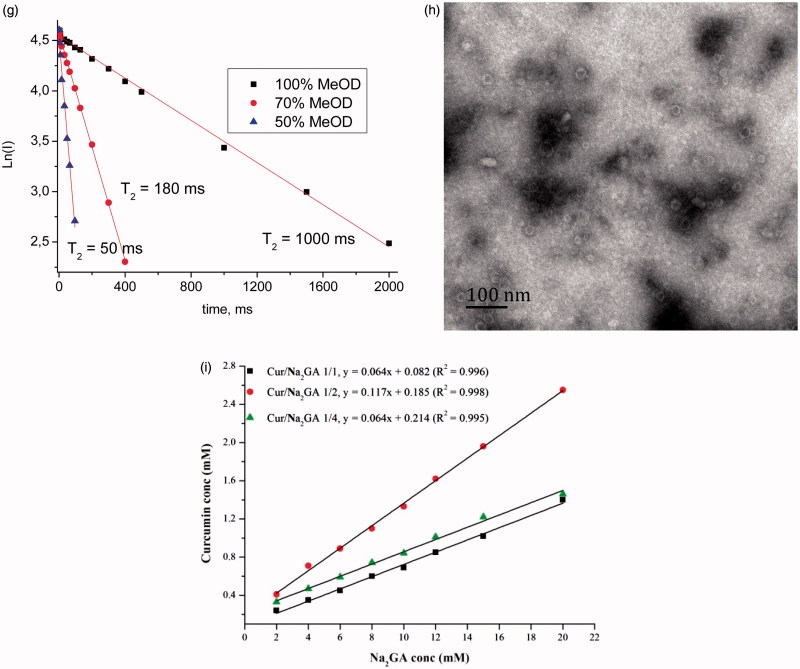

^1^H NMR spectra were used to further investigate the mechanism of Cur self-assembling. Figure 2(c) shows the ^1^H NMR spectra of Cur in different solvents. It could be seen that after the addition of water, a sharp decrease in the NMR peaks intensity of Cur molecules occurred, due to solubility decrease. Self-assembling of Cur was confirmed by measurement of *T*_2_ relaxation time of Cur. The experiment showed the reduction of the relaxation time of Cur protons (Figure 2(d)) with increasing of water content in water/methanol mixtures. The NMR results demonstrated that self-assembles of Cur should have sufficiently lower diffusion mobility compared with single molecules, which lead to a considerable broadening of the lines and shorten the time of the spin–spin relaxation (Poole, 1971; Deese et al., 1982; Kornievskaya et al., 2007; Polyakov et al., 2008; Selyutina et al., 2016b). The change of chemical shifts of Cur protons and decrease of relative intensity of 6-H proton with increase of water content could be also related to keto-enol tautomerism.

Actually, we can evaluate, that broadening of lines and shortening of *T*_2_ can occur more than at least 10 times. The precise measurements are practically impossible because very low concentration of Cur in pure water solutions. The noted values correspond to lowering of rotational diffusion mobility to nearly same times. Taking in mind, that according to well-known Stocks–Einstein equation, *D*_rot_∼1/*d* ^3^ we can apologies that volume *V*∼*d* ^3^ of cluster should exceed the same value for single molecule at least in 10 folds. So our data are not in contradiction with theoretically predicted cluster dimensions.

Obtained results on self-assembling of Cur and its tautomer transformation in different environment were prominent not only for understanding of structure of its solution, but for choosing of the right method for quantitative analysis of its concentrations. In our opinion, it is more correct to use the technique of reverse-phase HPLC, which enables to detect all the amounts of Cur in the solution, regardless of the degree of tautomerism and self-assembling.

Solubility and dissolution profiles of free Cur, (PMs), and Cur SDs are shown in Figure 2(e–f). It could be seen that there was no significant differences between free Cur (1.73 mg/L) and PMs (1.98–10.87 mg/L). However, Cur SDs exhibited excellent solubility (723.58 mg/L, >400 times) compared to free Cur and PMs, affording better results with higher content of Na_2_GA in longer milling time. It was supposed that amorphous SD provided better wettability and dispersibility as the drug was in its supersaturated state due to forced solubilization in the hydrophilic carriers. The formation of micelles gave rise to excellent solubility of Cur/Na_2_GA SD. In contrast, PMs with low amorphous state and heterogeneous dispersion could not achieve good solubility. We can assume the following mechanism of this effect. There are two competing processes in the dissolution of SD and PM, the self-association of Cur and the association of Cur with Na_2_GA. When the PM dissolves, the self-association prevails, and upon dissolution of the SD, the Cur-Na_2_GA associates initially exist, which leads to an increase in solubility. In case of the stability, the Cur contents in samples with milling time of 24 h were more than 95%, suggesting there was no significant destruction and loss for Cur during mechanical treatment. It could be readily deduced from Figure 2(f) that the Cur SDs showed fast dissolution rate in pH 6.8 phosphate buffer. The cumulative amount of Cur dissolved after 15 minutes was 27.7%, 47.8%, and 74.0% for SDs with Cur/Na_2_GA molar ratio of 1/1, 1/2, and 1/4, respectively. In contrast, slight amount of Cur was dissolved in pH 1.2 simulated gastric media. Therefore, Cur SDs with milling time of 24 h were chosen to be further investigated concerning their excellent solubility and dissolution rate.

### 3.3. Characterization of Cur/Na_2_GA micelles

As shown in Table 1, gel-permeation HPLC was used to analyze the MWD of Cur/Na_2_GA SDs. Previous studies (Dushkin & Tolstikova, 2012) revealed that glycyrrhizin acid (GA) dissolved in water could form micelles with molecular weight of 66 kDa, composed of approximately 80 GA molecules. Na_2_GA had the same property as GA that formed high molecular weight of 85–90 kDa when dissolved in water, approximately 100 molecules. According to studies (Kornievskaya et al., 2007; Polyakov, 2011), critical micelle concentration of glycyrrhizin is near 1 mM. It was assumed that the use of the sodium salt of GA did not prevent the formation of micelles, since this compound could undergo hydrolysis in aqueous solutions and formed a free GA. Cur/Na_2_GA SD with milling time of 24 h had a little larger molecular weight than that of 2 h, probably due to long-time milling promoted Cur molecules to be much more homogeneously dispersed in Na_2_GA molecules and facilitated the formation of micelles.

**Table 1.** Molecular weight distribution^a^, size and zeta-potential of Cur SD in water solution.

Samples	Cur/Na_2_GA, 1/1	Cur/Na_2_GA, 1/2	Cur/Na_2_GA, 1/4
*M*_n_ (kDa), 2 h/24 h	83.7/85.0	85.0/87.9	88.3/88.5
*M*_w_ (kDa), 2 h/24 h	85.9/87.1	87.2/90.2	90.6/90.8
*M*_p_ (kDa), 2 h/24 h	79.3/80.9	80.6/84.8	84.8/84.8
Size (nm), 2 h/24 h	43.83 ± 2.2/85.40 ± 4.5	46.20 ± 0.8/91.9 ± 6.0	71.4 ± 0.7/135.3 ± 7.9
PI, 2 h/24 h	0.272 ± 0.020/0.161 ± 0.013	0.241 ± 0.028/0.312 ± 0.001	0.319 ± 0.011/0.252 ± 0.009
Zeta-potential (mV), 2 h/24 h	–37.7 ± 0.7/–38.6 ± 2.2	–37.6 ± 0.6/–38.9 ± 1.6	–39.6 ± 1.2/–43.5 ± 0.9
Thermodynamic values (+37 °C, *T* = 310)			
Slope	0.064 ± 0.0024	0.118 ± 0.0019	0.064 ± 0.0018
*S*_0_	0.096 ± 0.026	0.188 ± 0.021	0.214 ± 0.020
*K*, M^–1^	703.6 ± 195.4	713.2 ± 81.8	320.1 ± 30.6
Δ*G*, kJ/mol	–16.9 ± 0.7	–16.9 ± 0.3	–14.9 ± 0.2

^a^ *M*_n_: number average molecular weight; *M*_w_: weight average molecular weight; *M*_p_: molecular weight at peak maximum of the elution diagram.

^1^H NMR spectroscopy was also applied for investigation of the incorporation of Cur into the micelles of glycyrrhizin in different environment. As shown in Figure 2(g), in the presence of Na_2_GA in aqueous-methanol solution, there was a substantial shortening of *T*_2_ relaxation time of O–CH_3_ protons due to lowering of diffusion mobility of molecules. Since it was known that GA did not form micelles at methanol content >40% (Kornievskaya et al., 2007), we could suggest that in the system under study the aggregation of Cur/Na_2_GA complexes should be accomplished by formation of micelles with incorporated molecules of Cur.

The size and surface morphology of micelles are of great importance for interactions between the cell membranes and micelles. The size and size distribution of Cur SD self-formed micelles were determined by dynamic light scattering (DLS), which showed a monomodal particle size distribution with a mean diameter of 43.83–71.40 nm for milling time of 2 h (Table 1) and 85.40–135.3 nm as milling time prolonged to 24 h. These results were consistent with the MWD data, that long-time milling facilitated the formation of a more homogeneous dispersion that enabled large amount of Cur molecules to be embedded into the micelles and therefore enlarged the micelles size. Generally, the zeta potential above ±30 mV could form a physically stable dispersion. As shown in Table 1, high zeta-potential (37.6–43.5 mV) of the micelles might have contributed to their stability.

The TEM images and visual appearance of micelles are depicted in Figure 2(h). Micelles displayed a spheroid shape with smooth boundaries and particle diameter ∼30 nm. The decrease of the size compared to DLS data probably resulted from the shrinkage of the micelles when dried before TEM analysis.

Phase solubility studies for Cur/Na_2_GA systems were carried out in aqueous systems at different temperatures to calculate the stability constants (*K*_s_) and the thermodynamic values for the formation of supramolecular micelles systems. Unfortunately, the experimental data for temperatures below +37 °C had large errors because of the possible instability of SD – water micelle solution equilibrium. The typical observed diagrams for different Cur/Na_2_GA relations and concentrations are shown in Figure 2(i). They represented a standard liner (A_L_-type relationships), suggesting the formation of 1:1 ‘molecular relations’ of associated complexes/micelles (Higuchi & Connors, 1965). Thus, in the solubility diagram, Cur and Na_2_GA appeared as fixed molecular ratio to form micelle. Given the very narrow MWD of the micelle (see Table 1), this assumption might be well founded. The slopes of obtained isotherms were less than 0.12, proving the molecular stoichiometry of the complex/micelles was one to one, too.

Additionally, the equilibrium constant (*K*_s_) of this system was calculated according to Equation (1). All results of calculation using Equation (1) and (2) are displayed in Table 1. The values of stability constant *K* and Δ*G* of Na_2_GA/Cur (1/1 and 2/1) were nearly the same. But increasing content of Na_2_GA to Cur (4/1) decreased the stability constant *K* and Δ*G*, which probably suggested that weaker inclusion of Cur molecules into micelles of GA. Additionally, Δ*H* values for all samples were negative, and their exact values were not shown due to the large estimated error, as mentioned previously. Therefore, the thermodynamics parameters indicated that the formation of this inclusion complex could spontaneously complete (Δ*G* < 0), and the inclusion process was exothermic (Δ*H* < 0).

### 3.4. Cytotoxicity assay

Curcumin has been extensively studied in modern medicine and Indian systems of medicine for the treatment of various medical conditions, including breast cancer. Recent research revealed that Cur might be valuable in the prevention and therapy of neurodegenerative diseases like Alzheimer's disease and also inhibiting the formation of brain tumors. Due to good lipophilicity, Cur can easily penetrate endothelium and reach tumor tissue in mammary gland, also Cur can cross blood–brain barrier and reach the brain tissue (Mishra & Palanivelu, 2008; Tsai et al., 2011; Hagl et al., 2015). According to this, we used common cell line of breast cancer (MCF-7) and human primary glioblastoma cell line (U-87 MG) to investigate cytotoxic activity of pure Cur and Cur SDs by MTT assay. Immortalized human fibroblasts cell line was also used and served as a model of non-tumor tissue.

**Figure 3.**
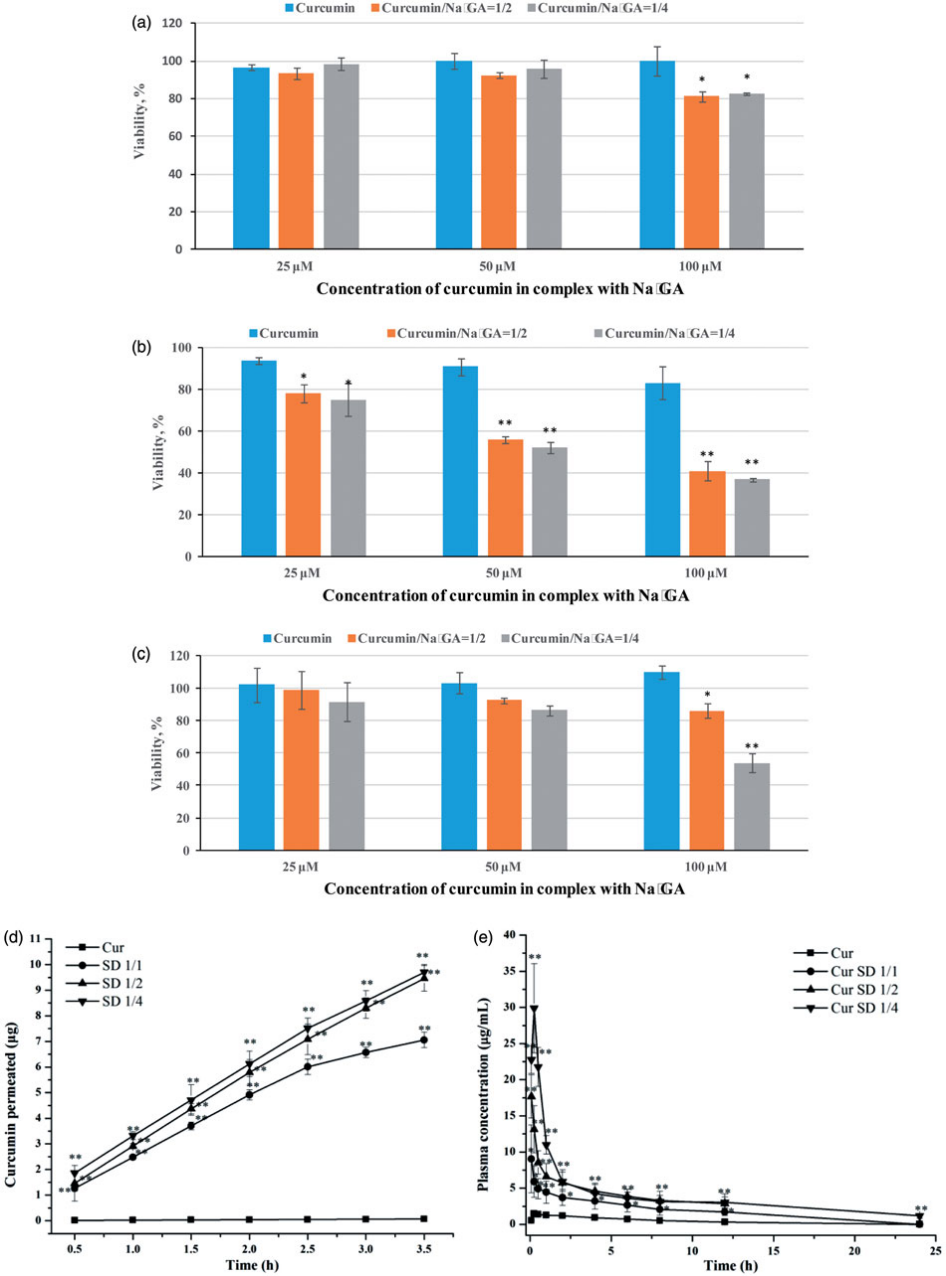
Cytotoxic effects of pure Cur and Cur SDs on (a) MCF-7 cells; (b) U-87 MG cells; (c) immortalized human fibroblast cells, results are presented as mean viability ± SEM; (d) permeation profile of pure Cur and Cur SDs in PAMPA experiment; (e) concentration of curcumin in rat plasma after a single oral dose of 150 mg/kg of free Cur or Cur SD, *p < .05 compared to pure Cur in same point, **p < .01 compared to pure Cur in same point, values are mean ± SEM (*n* = 5).

To determine the cytotoxic activities of pure Cur and Cur SDs, cytotoxicity studies were performed on human glioblastoma U-87 MG, breast cancer MCF-7 and immortalized human fibroblasts cell lines by MTT assay. Figure 3(a–c) shows cell viability of cell lines after 48 h of incubation time. It was shown that complex formation with Na_2_GA increased cytotoxic properties of Cur in dose-dependent manner. The effect was most pronounced in human glioblastoma cell line. As shown in Figure 3(b), mean cell survival % treated by Cur SDs with Cur concentration range of 25–100 μM decreased from 76 to 37%, whereas cell survival % treated by free Cur in same concentration range stayed above 80%. It was important that this cytotoxicity against glioblastoma cells was specific because no significant cytotoxicity of Cur SDs in immortalized human fibroblasts at concentrations 25 and 50 μM was found. In these cells, Cur/Na_2_GA 1/4 at concentration of 100 μM possessed more prominent cytotoxicity compared to Cur/Na_2_GA 1/2. All obtained cytotoxic action of Cur SDs was due to the Cur effect. As shown in Table 2, Na_2_GA itself possessed no cytotoxicity in all tested cell lines in concentration range of 25–400 μM.

**Table 2.** Cytotoxicity of Na_2_GA in MCF-7, U-87 MG and immortalized human fibroblasts cell lines.

	Viability, %		
Concentrationof Na_2_GA, μM	MCF-7	U-87 MG	Immortalized human fibroblasts
25	98.2 ± 2.13	96.6 ± 2.39	96.2 ± 2.78
50	97.8 ± 3.33	95.9 ± 1.16	97.3 ± 1.73
100	98.2 ± 0.41	92.2 ± 2.75	98.8 ± 4.18
200	98.7 ± 1.59	92.2 ± 2.63	96.0 ± 1.66
400	99.6 ± 1.34	92.9 ± 1.65	98.3 ± 0.90

Values are presented as mean viability ± SEM.

Thus, the important finding of cytotoxicity studies was that the formulation of Cur SDs improved cytotoxic capacity of Cur. The greater cell inhibition of the Cur SDs might be attributed to high cell uptake. *In vitro* environment, free Cur would diffuse throughout the intracellular environment via the passive diffusion mechanism. Due to the poor solubility and therefore the low concentrations of Cur in the local media, it would not be significantly for the uptake. In contrast, Cur loaded micelles could not only enhance water solubility and prevent Cur from assembling or degradation, but also might result in higher cell membranes permeability and therefore better cell cytotoxicity.

### 3.5. Permeability and bioavailability studies

The PAMPA enabled fast determination of the trends in the ability of the compounds to permeate membrane by passive diffusion and was thus suitable for screening potential drugs. Results (Figure 3(d)) showed a strong increasing of the amount of Cur permeated in comparison with a saturated aqueous solution of pure Cur used as control. In the case of Cur SD with the molar ratio of 1/1, 1/2, and 1/4, permeation could reach 7.05 μg, 9.46 μg, and 9.70 μg, respectively, during 3.5 h, suggesting higher ratio of Na_2_GA in SDs could promote Cur to faster membrane permeation. According to our previous NMR and molecular dynamics studies (Selyutina et al., 2016a,b), glycyrrhizin molecules are able to penetrate into lipid membranes and change their physical and functional properties, including permeability. The obtained results suggest a sharp increase in bioavailability of Cur from obtained Cur SD during experiments *in vivo*.

Figure 3(e) depicts the respective concentration vs. time curve of Cur in rat plasma after oral dose of 150 mg/kg of either Cur SDs or free Cur, and the pharmacokinetic parameters are summarized in Table 3. It could be seen that high ratio of Cur/Na_2_GA afforded better bioavailability of Cur. Cur SD with Cur/Na_2_GA molar ratio 1/4 demonstrated a ∼19-fold increase in AUC and a ∼20-fold increase in *C*_max_ concentration compared to free Cur. The concentration of Cur in plasma increased to the highest levels (29.87 μg/mL) after administration of 15 min and then decreased gradually. The increase in bioavailability of Cur SD may be attributed to several reasons. First, it was well accepted that the oral bioavailability of lipophilic compounds increased in the following order: powder, suspension, and solution (Veber et al., 2002). Na_2_GA enhanced the solubility of Cur by forming micelles and aided the formation of a homogenous solution. Second, the crystalline vs. amorphous nature of free Cur and Cur SD, respectively might play a role. Amorphous SD provided better wettability and dispersibility as the drug was in its supersaturated state due to forced solubilization in the hydrophilic carriers. The increased solubility gave improved bioavailability.

**Table 3.** Pharmacokinetic parameters of curcumin following oral administration of free Cur and Cur SD.

Parameters	Free Cur (control)	Cur SD 1:1	Cur SD 1:2	Cur SD 1:4
*C*_max_ (μg/mL)	1.48 ± 0.03	9.04 ± 0.35**	17.68 ± 0.22**	29.87 ± 2.71**
*T*_max_ (min)	15 ± 0.00	5 ± 0.00	5 ± 0.00	15 ± 0.00
*T*_1/2_ (h)	5.07 ± 0.41	8.02 ± 0.70**	9.05 ± 0.65**	11.16 ± 1.03**
AUC_0→_*_t_* (μg/mL.h)	12.12 ± 0.36	64.50 ± 3.12**	108.16 ± 5.2**	231.61 ± 10.15**
AUC_0→inf_ (μg/mL.h)	14.26 ± 0.72	104.38 ± 6.21**	181.60 ± 8.76**	279.50 ± 12.11**

AUC: area under the plasma concentration–time curve; *C*_max_: peak concentration; *T*_max_: time to reach peak concentration.

Values are reported as mean ± SEM (*n* = 5).

** *p* < .01 compared with the control.

Third, it was reported that the increased permeability of drug by GA was not only due to increased solubility, but also to the enhancement of drug permeability passing through cell membranes (Polyakov, 2011; Polyakov & Kispert, 2015). NMR indicated pore formation in the presence of GA and it could penetrate into the lipid bilayer, which predominantly located in the outer ‘half-layer’ of the liposome and the middle of the hydrophobic tails was the preferred location. Then, GA changed the mobility of lipids and freely passed through the bilayer surface to the inner part bringing some water molecules (Selyutina et al., 2016b). Na_2_GA had the similar properties as GA and could act as a good biologically active compound and drug carrier.

## 4. Conclusions

In the present investigation, an amorphous Cur SD using ball milling approach was successfully prepared. Cur SD exhibited superior solubility characteristics as compared to free Cur. Consistent with the amorphous nature and self-formed micelles of Cur SD, a marked improvement in pharmacokinetic behavior evidenced by a ∼19-fold increase in oral bioavailability was demonstrated in rats. The significant increase in bioavailability was also corresponding to the increase in bio-efficacy and membrane permeability, with Cur SD demonstrating greater cytotoxic activity than free Cur. Employing Cur SD will allow the use of lower doses while maintaining efficacy and may also help overcome some of the disadvantages of color, taste and smell. Although there have been other reports on using SD to improve the bioavailability of Cur, our work illustrated an unprecedented preparation of an amorphous Cur SD formulation by mechanical ball milling and the significant innovation to form self micelles.
